# Five strategies on writing research papers for beginners and young general medicine doctors

**DOI:** 10.1002/jgf2.603

**Published:** 2023-01-18

**Authors:** Taiju Miyagami, Kosuke Ishizuka, Taku Harada, Hiroyuki Nagano, Yuki Otsuka, Tomoko Kumakawa, Shun Yamashita

**Affiliations:** ^1^ Department of General Medicine, Faculty of Medicine Juntendo University Tokyo Japan; ^2^ Division of General Internal Medicine, Department of Internal Medicine St. Marianna University School of Medicine Kanagawa Japan; ^3^ General Medicine Nerima Hikarigaoka Hospital Tokyo Japan; ^4^ Department of Healthcare Economics and Quality Management, Graduate School of Medicine Kyoto University Kyoto Japan; ^5^ Department of General Medicine Okayama University Graduate School of Medicine, Dentistry and Pharmaceutical Sciences Okayama Japan; ^6^ School of Public Health University of California Berkeley California USA; ^7^ Department of General Medicine Saga University Hospital Saga Japan

## Abstract

We propose five important strategies for young generalists to write original research and papers. We hope that even beginners will understand and practice these five strategies, and help young generalist to write research papers based on clinical questions that arise in their daily practice.

To the Editor:

Japanese general medicine doctors (GMs) tend not to conduct research and place importance on it compared with clinical practice and education.^1^ Tips for GMs to publish case reports emphasize the importance of clarifying the blueprint of the writing process in advance.^2^ However, there are no reports on methods for publishing clinical research papers, except for case reports. We consider that for GMs to conduct research, strategies should be clarified, including those aspects which they share with specialists. Seven GMs (median years since graduation: 10 years), who have actively published original papers in international peer reviewed journals, completed interactive interviews and a narrative literature review about the likely stumbling blocks for beginners (who have never written a paper before) while writing their first online research paper. Hence, we propose five important strategies to help young GMs author original research papers. (Table 1).

**TABLE 1:** Five strategies for becoming an academic generalist

Strategy 1. Keeping the motivation.
Strategy 2. Connecting clinical question to research question on a daily basis.
Strategy 3. Reviewing previous research.
Strategy 4. Conducting descriptive studies first.
Strategy 5. Assigning a mentor and forming a project team.

## STRATEGY 1: KEEPING THE MOTIVATION

First, being motivated in any situation and challenging daily practice makes the emergence of a clinical question more likely. The joy we experience when our products are published and by knowing that having a research perspective improves our clinical skills is motivation in itself. We encourage GMs who have not yet taken the plunge to take the first step.

## STRATEGY 2: CONNECTING CLINICAL QUESTIONS TO RESEARCH QUESTIONS ON A DAILY BASIS

To conduct research, it is crucial to link clinical questions—questions that occur in the clinical setting—to research issues by considering their ethics, novelty, and viability. Eventually, we may be able to form GMs' original research team.

## STRATEGY 3: REVIEWING PREVIOUS RESEARCH

We regret not conducting a thorough review of previous studies during the planning stage, either due to a lack of research items after beginning research or to previously published reports on the subject. Conducting a generic, yet comprehensive search of previous studies and setting survey items and inclusion criteria are critical.

## STRATEGY 4: CONDUCTING DESCRIPTIVE STUDIES FIRST

This strategy is particularly important among these five strategies. Descriptive studies are able to summarize study data efficiently and logically, without a control group, intervention, or complex statistical calculations.^3^ Investigating epidemiological frequencies enables answering questions in clinical practice. In addition, GMs have a high affinity for descriptive research because they have conducted several studies on diagnosis and epidemiology.^4^ In our experience, we recommend that beginners start with descriptive studies.

## STRATEGY 5: ASSIGNING A MENTOR AND FORMING A PROJECT TEAM

Hospitalists in the U.S. report that the lack of a mentor interferes with their research work.^5^ Therefore, finding a mentor to discuss team building in detail and determining the division of roles between the mentor and the team are crucial. Mentors may not be easily available at GMs' own institutions; thus, it is important to find one at academic conferences or social network services and actively seek advice.

We hope that these five strategies will help beginners and young GMs author research papers.

## AUTHOR CONTRIBUTIONS

All authors had access to the data and a role in writing the manuscript.

## CONFLICT OF INTEREST

None.
